# Upper Respiratory Tract Symptoms, Renal Involvement and Vasculitis: A Case Report and Review of Wegener Granulomatosis

**DOI:** 10.4021/jocmr412w

**Published:** 2010-08-18

**Authors:** Mohd Shahrir Mohamed Said

**Affiliations:** University Kebangsaan Malaysia Medical Centre, Jalan Yaacob Latif, Cheras 56000 Kuala Lumpur, Malaysia. Email: drobiwan@gmail.com

## Abstract

Wegener’s Granulomatosis is a condition associated with systemic vasculitis which can present with upper respiratory tract symptoms initially. On September 2001, a 15-year-old girl presented with symptoms of nasal block for 3 weeks. She later developed joint pains and worsening renal status requiring dialysis. A renal biopsy was performed which showed pauci-immune cresentric glomerulonephritis. Her cANCA levels were positive. She was treated with oral cyclophosphamide and steroids and later responded.

**Keywords:**

Wegener granulomatosis; Young girl; Cyclophophamide; cANCA

## Introduction

Wegener’s Granulomatosis (WG) is a condition associated with generalized vasculitis and was first reported by Heinz Klinger and Frederick Wegener. Appropriately, Wegener described the lesion as ‘rhinogenic granulomatosis with special involvement of the arterial system and the kidneys’. The peculiar aspect of this case was that the patient was a girl who was only 15 years of age. In fact there was only one case report so far of WG in a 10-year-old girl. The diagnosis was clinched by the typical renal biopsy.

## Case Report

Miss CYY was a 15-year-old schoolgirl who was referred from Fatimah Hospital. She first presented with a three week history of bilateral nasal block. She then developed joint pains. She also developed oral ulcers and maculopapular rashes over her left elbow, left ankle and left popliteal fossa. Her eyes became red and watery with a clear discharge. She also complained of photophobia and insomnia and gastrointestinal symptoms of anorexia and nausea. However, there was no pruritus, vomiting or change of behaviour.

On examination, she was pale, with minimal ankle oedema. Jugular venous pressure was not raised. There were oral ulcers and her eyes revealed injected conjunctiva but no hemorrhages. Eye examination revealed sluggish papillary reflex, injected sclera and haziness over the anterior chamber. Cardiovascular, respiratory and abdominal examinations were all essentially normal. There was a purpuric rash over her right elbow, left ankle and left popliteal fossa.

Her full blood count revealed an Hb of 7.6 g/dL, total white cell count of 27,800 /ml and a platelet count of 840,000 /ml. ESR was 138 mm/hr. C reactive protein was elevated at 199 mg/L. On admission, blood urea was 16.5 mmol/L which progressively increased to a level of 46.8 mmol/L. Se Creatinine was 680.6 mmol/L. Urine examination revealed numerous red blood cells and no albuminuria. There were no red cell casts and white cell casts. No urine phase contrast was done. The cytoplasmic ANCA was positive. Anti dsDNA and ANF were negative. Serum C3 and C4 were low. Hepatits BsAg was negative and so was the HIV serology. Both Coombs tests direct and indirect were negative. Oesophago-Gastro-Duodenalsopy (OGDS) revealed multiple hemorrhagic spots of the gastric walls. No gastric biopsy was done. VDRL was negative. Spiral CT scan of the sinuses revealed bilateral maxillary and ethmoid sinusitis.

Renal biopsy following adequate dialysis revealed fibrocellular and sclerotic crescent global involvement. There were 28 glomeruli. Among them 24 glomeruli showed partial crescent formation with compressed glomerular capillary loops. There was also extension of the fibrosis into periglomerular tissue on silver staining. There were no intracapillary or granuloma formation. The tubules showed degenerative and regenerative changes with occasional white blood cell cast. The interstitium was edematous with a dense inflammatory infiltrate consisting of lymphocytes, plasma cells, histiocytes and neutrophils. Immunoflourescent study revealed weak segmental glomerular capillary wall staining with IgG, IgM and C3. IgA was negative. Blood vessels showed no reactivity. The histologic diagnosis was pauci-imunne cresentric glomerulonephritis consistent with WG.

She was pulsed with intravenous methylprednisolone 250 mg daily for 3 days followed by oral prednisolone and intravenous cyclophosphamide. Later, oral cyclophosphamide was started. She responded well to treatment.

## Discussion

The term ‘systemic vasculitis’ can occur as a primary event, e.g. in the ANCA associated vasculitides, or secondary to other established diseases (secondary vasculitis), e.g. collagen vascular disorders. The classification of vasculitides was developed by the Chapel Hill Conference in 1992 [1] and was based on: (1) clinical and histopathologic features; (2) the size of the predominant vessel involved; (3) the presence of serologic markers and other immune phenomena; (4) and/or the affected tissue (for exemple, immune deposits), as demonstrated by immunohistochemistry.

There are four types of hypersensitive reactions according to Coombs and Gell: (I) immediate hypersensitivity; (II) antibody mediated hypersensitivity; (III) immune complex mediated hypersensitivity; and (IV) T-cell mediated hypersensitivity. Vasculitis is a type II reaction [1].

There are three types of anti neutrophil cytoplasmic antibodies (ANCA) known currently. The first one is the classic granular cytoplasmic pattern (cANCA). The second is the perinuclear fluorescence pattern (pANCA). And the third is the atypical ANCA (aANCA) [1].

In WG, cANCA gives a 95% specificity. However, its level depends also on the extent and phase of the disease. In the initial phase whereby WG is localized to the upper and lower respiratory tracts, cANCA confers a 50% specificity. In the generalized form the specificity is 100%. In complete remission cANCA is not detectable.

The hallmark of WG includes the clinical feature of pulmonary-renal vasculitis, immunohistochemical (pauci-immune vascultis or glomerulonephritis) and serologic markers (cANCA). Renal biopsy would show a necrotizing (GN) with crescent formation as typically depicted by this case report. Immunohistochemistry studies of the renal tissue reveals a pauci-immune picture. However, in Goodpastures syndrome and Henoch-Schonlein purpura there are linear IgG deposits in the capillary and mesangium along glomerular basement membrane and IgA deposits respectively [1].

WG is associated very closely with an enzyme called Proteinase 3 (PR3). PR3 is stored in the azurophilic granules of human polymorphonuclear leucocytes and monocytes and serve as autoantigens for vasculitis associated ANCA. PR3 also controls growth and differentiation of leukaemic cells, micorbacteriacidal activity and activation of cytokines.

ANCA is a pathogenic factor in inflammatory processes that underlie necrotizing vasculitis. There are four hypotheses to the pathogenensis of ANCA in vasculitis process: (1) The first hypothesis states that it is responsible for interacting with effector cells and rarely interferes with physiologic functions of the target antigen [1]. (2) The second hypothesis states that ANCA binds to PR3 reducing its proteolytic activity while at the same time preventing immune complex formation and complete inactivation by alpha-1-antitrypsin. Dolman et al [2] postulate that the inhibitory effect of ANCA on PR3-alpha-1-anti-trypsin complex formation correlates with clinical activity in WG patients [2]. (3) The third postulates that ANCA can activate primed PMN to release oxygen radicals and lysosomal enzymes [3, 4]. (4) The fourth is known as the ANCA-cytokine sequence theory [5]. It proposes that priming doses of proinflammatory cytokines induce surface expression of ANCA target antigens. Binding of ANCA to these antigens leads to full neutrophil activation and endothelial cell injury and subsequent vascular injury. It was demonstrated in vitro and ex vivo by flow cytometry that TNF alpha and Interleukin 8 act synergistically to induce a translocation of PR3 from intragrannular loci to the cell surface of PMN. In murine models, it was demonstrated that monoclonal ANCA IgG binds to PR3 and engages Fc-gamma-RIIa and activate PMN via receptor mediated signal transduction system.

In active ANCA associated glomerulonephritis resident cells, endothelial cells and infiltrating mononuclear cell express cytokines, (Interleukin 1, Interleukin 2, Tumor Necrosis Factor alpha, Interferon gamma, Platlet Derived Growth Factor and Tumor Growth Factor beta), growth factor receptors, (Tumor Necrosis Factor receptor, Interleukin 1 receptor type II, Interleukin 2 receptor, Interferon receptor and Platlet Derived Growth Factor beta receptor), and adhesion molecules, (ICAM-1 and ELAM-1) [4, 5].

Renal lesions in WG demonstrate the abundance and intensity of expression of ICAM-1 which correlate with the presence of glomerular crescents and the number of LFA-1 positive (CD11a) leucocytes. Thus, the serum levels of these soluble adhesion molecules correlate with disease activity and renal impairment [5].

Genetically, WG has some correlation with HLA class II. Presence of ANCA correlates with presence of DQw7 and DR4 haplotypes. The cANCA is associated with increased frequency of the Z allele [6].

Coxsackie B3 and Parvovirus-B19 had been postulated as environmental triggers of ANCA and /or WG. Stegemann et al [7] had reported association of chronic nasal carriage of Staphylococcus aureus with higher relapse rates in WG.

In a study conducted on 477 WG patients it was found 23 of these patients had malignancies. Seven out of 23 had renal cell carcinoma [1].

As for drug induced ANCA associated vasculitis, propylthiouracil and hydralazine were known causes for this phenomenon.

There were many theories which try to explain the basis of autoimmune diseases. One such popular theory is the idiotype-anti-idiotype reactions. It was believed that autoantibody formation is regulated by this idiotype-anti-idiotype reactions network. Defect in the network results in production of autoantibodies [8, 4].

According to Hoffman et al [9] and Barth et al, airway stimulation form a variety of sources triggers a neutrophilic alveolar response in predisposed patients to develop WG and to develop ANCA, which may result in enhanced recruitment and activation of monocytes, lymphocytes and eosinophils. However, no specific sources were actually identified.

At presentation 80% of WG patients have no renal involvement. However, when patients present with destructive, sterile inflammatory lesions of the upper and lower respiratory tract and glomerulonephritis, WG must always be ruled out. In 50% of these patients there could be a 3 to 6 months delay in diagnosis. About 73% of these patients will present with nasal, sinus, tracheal and/or ear abnormalities. Often, these complaints will be attributed to upper respiratory infections or allergy. Recurrent epistaxis, mucosal ulcerations, nasal septal deformities or perforations will lead to a more extensive evaluation. Other ENT symptoms include: sinusitis, nasal diseases, otitis media, hearing loss, subglottic stenosis, ear pain and oral lesions, all in decreasing frequency. Lung presentation includes: infiltrates, nodules, hemoptysis and pleuritis. Eye diseases include: conjunctivitis, dacrocystits, scleritis, proptosis, eye pain, visual loss, retinal and corneal diseases also in decreasing frequency. Only 18% will present with glomerulonephritis. However, 75% of WG patient will eventually develop GN [1].

Abnormal investigations such as red blood cells in urine, elevated erythrocyte sedimentation rate (ESR), anemia, chest X ray showing pulmonary infiltrates and positive cANCA may prompt the pursuit of a definitive tissue biopsy [1].

Increases in ESR (Westergren method) correlates with disease activity in 80% of patients [1]. Anemia is common with thrombocytosis. The principle target of cANCA is PR3. Numerous studies utilizing either indirect immunoflourescent (IIF) and/or enzyme linked immunassay (ELISA) have indicated that cANCA and positive ELISA PR3 reactions have a specificity for WG about 98%. When neutrophiles for IIF ANCA are fixed in formaldehyde the ANCA-related antigens remained in the cytoplasm. When fixed with ethanol three types of patterns emerged: cANCA, pANCA and aANCA [1].

Titres of cANCA correlates with disease activity. In a study by Boomsma et al [10], who did a study on prediction of relapses in WG by measurement of ANCA levels, they found that serial measurements of ANCA levels is valuable for early prediction of relapses in patients with WG. In a prospective study involving 100 patients with WG form 1996 to 1998, there were 37 patients who relapsed. Out of these 37 patients, 34 showed a rise in their ANCA levels [11].

In a prospective study done by Aasarod K et al [12], a renal hispathological study was done on 94 patients. Two biopsies were done, one at initial diagnosis and the other after 3 years. The study took 42.5 months and revealed segmental necrotizing glomerulonephritis and extracapillary proliferation, in 85.1% and 91.5% respectively in these biopsies. All 7.4% patients who had normal serum creatinine and urinary protein excretion less than 0.5g/day had crescents; and six out of 7.4% had segmental glomerular necrosis. This study also showed that renal biopsy is of value in defining renal involvement in WG, but it is of limited help in early stage of the disease and in predicting renal outcome. However, the second part of the conclusion was not valid because only 14% of the WG patients turned up for the second biopsy.

Differential diagnosies includes Churg-Strauss syndrome (CSS), angiocentric immunoproliferative lesions (AIL), Goodpastures disease and infective causes for destructive upper or lower respiratory tracts. In CSS the clinical features include asthma and marked eosinophilia. AIL includes angiocentric and angiodestructive infiltrates which are polymorphous and include atypical lymphocytes. Grade I AIL resembles WG but the renal involvement is interstitial nephritis and not GN. Goodpastures disease has anti glomerular basement membrane antibodies. Mycobacterium, fungi, actinomycosis and syphilis, squamous cell carcinoma, extranodal lymphoma and cocaine abuse can resemble local WG.

In this patient the management consisted of T. prednisolone 60 mg daily and oral cyclosphosphamide 100 mg od for six months. The aim of cyclophosphamide is to suppress the total white cell count to a level of 4,000 cells/dL. Patient was monitored monthly with full blood counts.

Current management of WG consists of oral cyclophosphamide (CP) 2 mg/kg/day in combination with oral prednisolone 1 mg/kg/day. Daily prednisolone is continued for four weeks and tailed down gradually over 1 to 2 months before converting it to an alternate day regimen. Then the dose is further tapered down till patient is solely on CP (1). The effect of CP is seen within 1 to 2 weeks. However, in severe disease CP is started at 3 - 5 mg/kg/day using the leucocyte count as a guide for dosage adjustments. Prednisolone is given from 2 to 15 mg/kg/day [1].

One dilemma about CP is the route of administration intravenous pulsed CP and oral CP. According to Haubitz et al [13] IV CP is just as effective as oral CP and associated with fewer side effects with ANCA associated vasculitis and renal involvement. Again this is only based on serum creatinines and not on biopsy results. In a study done by Guillevin et al [14], IV CP was as effective as oral CP but in the long term, treatment with IV pulse CP did not maintain remission or prevent relapses as effectively as oral CP. Compared to 13% of 23 patients on oral CP and GC, 59.2% of 27 patients on IV CP and GC relapsed. This study was a prospective multicenter randomized trial.

In a report involving pregnant WG patients, done by Luisiri et al [15], the patient was treated with GC and CP successfully. He also reported that there were 10 similar cases in literature and the disease can have a more aggressive course in pregnancy.

Other agents that have been used include: methotrexate (MTX), leflunomide, cotrimoxazole in localized WG, etanercept, mycophenolate, 15-deoxyspergualin, intravenous immunoglobulin, tumor necrosis factor, anti-lymphocyte directed therapy, anti-adhesion molecule directed therapy and immunoablation using high dose cytotoxic medication with or without stem cell rescue. There is also suggested sequential chemotherapy involving CP followed by azathrioprine and CP followed by methotrexate [11, 16-19].

In the study done by Langford et al [17], he used a combination of MTX and glucocorticoids (GC) in treating patients with renal outcome of WG. The study involved 42 patients of whom 21 developed GN. Baseline serum creatinine was 1.4 mg/dL. Twenty out of 21 patients achieved renal remission. After the follow up period, this study showed positive results for these patients. However, the study was too small to make any impact on the future management of WG. Furthermore, it did not monitor ANCA levels. There was also no renal biopsy follow-up. Langford et al [18] also conducted another study that used MTX as a remission maintenance regimen after using CP and GC as a remission induction. Of 31 patients, 16% relapsed. Again, this does not look promising. The studies with other agents involved very few patients.

Morbidity in WG occurs as a result of disease only, from the disease plus treatment and from the treatment only.

In the NIH study, permanent disease related morbidity included chronic renal insufficiency (42%), hearing loss (35%), nasal deformity (28%), tracheal stenosis (13%) and visual loss (8%). About 10% required dialysis. Renal transplant patients must be in remission before operation. Most of these patients achieved remission [1].

The only pathology associated with the disease plus treatment is progressive pulmonary insufficiency. There is no study done to prove this as yet.

Patients of CP may have hair loss (20%), CP cystitis (50%), bladder cancer (5%) and myelodysplasia (2%). Side effects related to GC include DM (8%), cataracts (21%), osteoporosis fractures (11%) and aseptic necrosis (3%) [1].

The malignancy rate of WG was compared to the National Cancer Institute Registry. There is an overall 2.4-fold increase in malignancy, a 33-fold increase in bladder cancer and an 11-fold increase in lymphoma. There was latency of seven months from the start of CP administration to the occurrence of transitional cell carcinoma of the bladder. The occurrence of atypical cells in the urine is suggestive of this phenomenon. A cystoscopy and appropriate biopsy should be carried out. If the tissue biopsy is normal then a 1 - 3 yearly cystoscopy should be carried out for surveillance [1].

Pneumocystis carinii Pneumonia (PCP) is a frequent complication in these patients. Of 180 patients 6% developed PCP. These patients all received CP treatment in the early phase and were lymphopenic. Chemoprophylaxis of PCP has been suggested [1].

All in all, the effective treatment of WG must include complete remission and prevention of relapses. Perhaps in the near future, when the pathogenesis of WG can truly be explained, we could develop a better agent for the treatment of WG.
